# S157. NEURAL CORRELATES OF SMOOTH PURSUIT EYE MOVEMENTS IN POSITIVE AND NEGATIVE SCHIZOTYPY

**DOI:** 10.1093/schbul/sby018.944

**Published:** 2018-04-01

**Authors:** Eliana Faiola, Maria F Urquijo, Inga Meyhöfer, Anna Kasparbauer, Maria Steffens, Michael Wagner, Nikolaos Koutsouleris, Ulrich Ettinger

**Affiliations:** 1 University of Bonn; 2 Ludwig Maximilian University Munich

## Abstract

**Background:**

Both schizophrenia patients and highly schizotypal individuals are known to perform worse in smooth pursuit eye movements (SPEM) as compared to healthy controls with low levels of schizotypy. However, little is known about the neural correlates of SPEM deficits in subjects with high levels of schizotypy. In a previous study, individuals with high total schizotypy levels showed reduced activation in motion processing and other visual areas during SPEM than low schizotypal controls. Interestingly, reduced activation in these areas is also observed in schizophrenia patients. This suggests that schizotypy and schizophrenia overlap not only at the behavioral, but also at the neural level. In the present study, we followed up on these intriguing results by differentiating between negative and positive schizotypal groups. We expected to find lower SPEM performance in both highly negative and highly positive schizotypes (HNS and HPS) as compared to low schizotypal controls (LS). Moreover, in the schizotypy groups, activation in the SPEM network was expected to be reduced in a similar way as previously reported for schizophrenia patients and highly schizotypal individuals.

**Methods:**

In this ongoing, bi-center study, 88 healthy subjects (28 HNS, 23 HPS, 37 LS) underwent functional magnetic resonance imaging (fMRI) at 3T during a smooth pursuit task with concurrent oculographic measurement. Sinusoidal targets with frequencies of 0.2 Hz and 0.4 Hz were presented in a block design, with pursuit blocks alternating with blocks of fixation.

**Results:**

At the level of performance, we found an interaction between target frequency and group for the root mean square error (RMSE) of eye position (p = .026). This result indicates greater performance detriments from low to high frequency in HPS, as compared to the other two groups. At the neural level, overall activations during pursuit across the entire sample were found in brain regions known to be part of the pursuit network, i.e. frontal and supplementary eye fields, lateral geniculate nucleus, and visual cortex including V5. However, none of these regions displayed activation differences between groups. With multiple regression analyses for each of the groups, we investigated associations between cluster peak voxel activations and performance. For the low target frequency, a negative association was found between visual area V5 in the left hemisphere and the total saccade frequency for HPS (β = –.462, p = .03). For the high target frequency, activation in left V5 was negatively associated with RMSE in the LS group (β = –.411, p = .01).

**Discussion:**

We replicated previous findings of reduced SPEM performance in highly schizotypal individuals. In addition, a negative association between activation in area V5 and RMSE was only found among LS, but not for the two schizotypy groups. This is in line with previous findings of SPEM impairments in schizophrenia patients being associated to alterations in motion sensitive area V5 and underlines the importance of motion processing for SPEM deficits in the schizophrenia spectrum. However, we also found an association between activation in V5 and total saccade rate during pursuit in HPS, suggesting that V5 abnormalities may be restricted to negative schizotypy. Given the relatively small number of participants in this analysis, we expect to find broader and clearer group differences of neural mechanisms during pursuit with larger sample sizes.

